# New Monocyclic Terpenoid Lactones from a Brown Algae *Sargassum macrocarpum* as Monoamine Oxidase Inhibitors

**DOI:** 10.3390/plants11151998

**Published:** 2022-07-31

**Authors:** Jaeyoung Kwon, Kyerim Lee, Hoseong Hwang, Seong-Hwan Kim, Se Eun Park, Prasannavenkatesh Durai, Keunwan Park, Hyung-Seop Kim, Dae Sik Jang, Jae Sue Choi, Hak Cheol Kwon

**Affiliations:** 1 Natural Product Informatics Research Center, Korea Institute of Science and Technology, Gangneung 25451, Korea; kjy1207@kist.re.kr (J.K.); klim8@korea.kr (K.L.); hoseong91@kist.re.kr (H.H.); seonghwankim0517@gmail.com (S.-H.K.); prasanna@kist.re.kr (P.D.); keunwan@kist.re.kr (K.P.); 2 Division of Bio-Medical Science & Technology, KIST School, University of Science and Technology (UST), Gangneung 25451, Korea; 3 KHU-KIST Department of Converging Science and Technology, Kyung Hee University, Seoul 02447, Korea; dsjang@khu.ac.kr; 4 Department of Biology, Gangneung-Wonju National University, Gangneung 25457, Korea; hskim@gwnu.ac.kr; 5 Department of Biomedical Science, Asan Medical Institute of Convergence Science and Technology, Seoul 05505, Korea; gogo1685@naver.com; 6 College of Pharmacy, Kyung Hee University, Seoul 02447, Korea; 7 Department of Food and Life Science, Pukyong National University, Busan 48513, Korea

**Keywords:** *Sargassum macrocarpum*, terpenoids, Sepbox, MAO enzyme inhibitor, molecular docking

## Abstract

Algae are unique natural products that can produce various types of biologically active compounds. The 70% ethanol extract of brown algae *Sargassum macrocarpum* collected from the East Sea of Korea inhibited human monoamine oxidases A and B enzymes (*h*MAO-A and *h*MAO-B) at a 50 μg/mL concentration. The bioassay-guided isolation was performed through solid-phase extraction and the Sepbox system followed by serial high-performance liquid chromatography on the reverse phase condition, resulting in the identification of two new monocyclic terpenoid lactones, sargassumins A and B (**1** and **2**). The planar structures of the compounds were determined by a combination of spectroscopic data. The absolute configurations were determined by the interpretation of circular dichroism data. Compound **1** exhibited mild *h*MAO-A inhibition (42.18 ± 2.68% at 200 μM) and docked computationally into the active site of *h*MAO-A (−8.48 kcal/mol). Although compound **2** could not be tested due to insufficient quantity, it docked better into *h*MAO-A (−9.72 kcal/mol). Therefore, the above results suggest that this type of monocyclic terpenoid lactone could be one of the potential lead compounds for the treatment of psychiatric or neurological diseases.

## 1. Introduction

Natural products such as plants and microorganisms have been the major sources of compounds employed in drug discovery and development. About two-thirds of all small-molecule drugs that have been approved in the past two decades were associated with natural products [1]. Algae are unique natural products that possess a rich content of minerals and organic substances. They have been mainly used as a profitable source of proteins, fats, carbohydrates, dietary fibers, and minerals [2]. However, recent studies have suggested that the regular consumption of algae can reduce the incidence of various diseases, including cancer and metabolic, degenerative, infectious, and cardiovascular disorders due to the presence of biologically active compounds [2]. In particular, *Sargassum* sp., brown algae distributed throughout the temperate and tropical oceans, contains various terpenoids, polysaccharides, polyphenols, sargachromenol, steroids, and plastoquinones, which have antioxidant, anticancer, anticholinesterase, anti-inflammatory, and immunomodulatory activities [3].

Human monoamine oxidases A and B *(h*MAO-A and *h*MAO-B) are isoenzymes that catalyze the oxidative deamination of monoamine neurotransmitters and dietary amines [4,5]. The degradation of these molecules is responsible for the normal functioning of synaptic neurotransmission, which leads to the proper regulation of emotional behaviors and other brain functions. However, these reactions produce harmful byproducts, including aldehydes and hydrogen peroxide, which are hallmarks of neurodegenerative diseases [4,5]. The overexpression of *h*MAOs can induce oxidation–reduction imbalances and mitochondrial damage, leading to neuronal and brain damage [6]. Hence, the inhibition of these enzymes has been one of the effective strategies for discovering antidepressant and neuroprotective molecules. So far, several molecules (isocarboxazid, phenelzine, selegiline, and tranylcypromine) have been clinically approved worldwide [7]. Among these drugs, the selective inhibition of *h*MAO-A is an effective approach for the treatment of psychiatric disorders, while selective *h*MAO-B inhibitors are those used for treating neurological disorders [8]. In addition, a considerable number of molecules have recently been discovered from a variety of sources, including natural and synthetic ones, to inhibit these enzymes. For example, phlorotannins such as eckol and dieckol derived from edible brown algae *Eisenia bicyclis* exhibited inhibitory activities against *h*MAO-A and *h*MAO-B, based on enzyme-based kinetics and molecular docking simulation [9].

**Figure 1:**
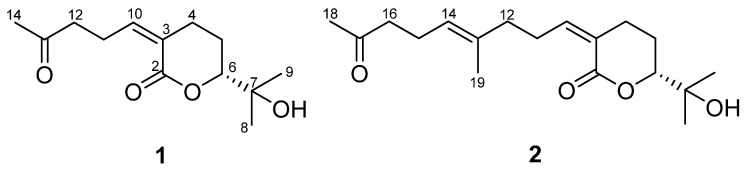
Structures of compounds **1** and **2**.

In the search for new inhibitors of MAO from algae, the ethanol (EtOH) extract of *S. macrocarpum* has been tested on *h*MAO-A and *h*MAO-B and has shown inhibitory activity on these enzymes at a 50 μg/mL concentration. This led to the isolation of two monocyclic terpenoid lactones (**1** and **2**) (Figure 1). These compounds were elucidated as new structures by a combination of spectroscopic and spectrometric techniques. This study reports on the isolation, structure characterization, and biological activities of the isolated compounds.

## 2. Results and Discussion

### 2.1. Bioassay-Guided Isolation of Compounds

The bioassay-guided fractionation of the ethanol (EtOH) extract of *S. macrocarpum* indicated that the dichloromethane (DCM) fraction (10, 40, and 200 µg/mL) showed inhibitory activities on human monoamine oxidases A and B *(h*MAO-A and *h*MAO-B) in a dose-dependent manner, while the aqueous fraction was not active (Table S1). A rapid chemical investigation of the biologically active DCM fraction was carried out using the Sepbox system. The major peak on the Sepbox chromatogram was deduced and found to be the previously reported known compound, sargachromanol A [10], as evidenced by the dereplication process utilizing the mass spectrum and the ultraviolet (UV) profile. Furthermore, two other peaks showed protonated molecular ions of *m*/*z* 241 and 309, respectively.

### 2.2. Structural Characterization of Compounds

**Figure 2:**
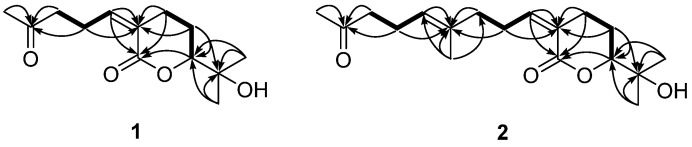
Key correlation spectroscopy (–) and heteronuclear multiple bond correlation (→) correlations of compounds **1** and **2**.

**Figure 3:**
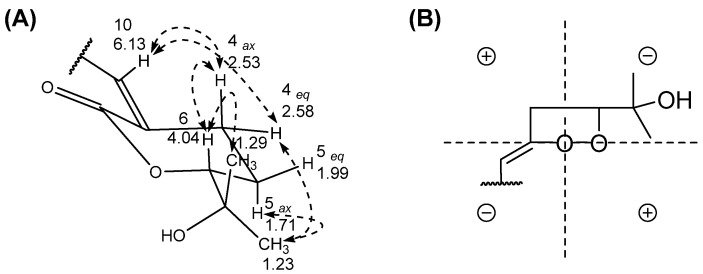
Key rotating frame overhauser enhancement spectroscopy correlations (**A**) and octant rule carbonyl projection (**B**) of compound **1**.

Compound **1** was isolated as a colorless oil, and the molecular formula was determined to be C_13_H_20_O_4_, based on high-resolution mass spectrometry (HRMS) data, indicating four indices of hydrogen deficiency. The ultraviolet (UV) absorption at 225 nm suggested that this compound lacked chromophores. The infrared (IR) absorption data displayed absorption bands at 3422 and 1695 cm^−1^, indicating the presence of hydroxy and carbonyl functional groups, respectively. The ^1^H nuclear magnetic resonance (NMR) data (Table 1) exhibited three methyl signals (*δ*_H_ 2.16, 1.29, and 1.23), four methylene signals (*δ*_H_ 2.83, 2.63, 2.58, 2.53, 1.99, and 1.71), an olefinic methine signal (*δ*_H_ 6.13), and an oxymethine signal (*δ*_H_ 4.04). The ^13^C NMR data (Table 1) exhibited 13 resonances comprising three methyl carbons, four methylene carbons, two methine carbons, and four non-protonated carbons. The presence of a double bond and two carbonyl carbons accounted for three out of four unsaturation equivalents, suggesting that this compound has a monocyclic ring, as evidenced by the correlation spectroscopy (COSY) correlations from H_2_-4 (*δ*_H_ 2.58) to H-6 (*δ*_H_ 4.04) via H_2_-5 (*δ*_H_ 1.99 and 1.71) as well as the heteronuclear multiple bond correlation (HMBC) correlations of H_2_-4/C-2 and C-3 and H_2_-5/C-3 and C-6 (Figure 2). Serial COSY correlations from H-10 (*δ*_H_ 6.13) to H_2_-12 (*δ*_H_ 2.63) indicated the presence of the linear fragment, and the HMBC correlation of H_3_-14/C-12 and C-13 implied that the acetyl group was present at the end of this linear fragment. Furthermore, the HMBC correlation of H_3_-8/C-6 suggested that the dimethyl carbinol group was attached at C-6. The rotating frame overhauser enhancement spectroscopy (ROESY) correlation H_2_-4/H-10 indicated that the geometry of the double bonds was 3*Z* (Figure 3A). Compound **1** was tentatively named sargassumin A.

Compound **2** was obtained as a colorless oil and the molecular formula was deduced and found to be C_18_H_28_O_4_, based on HRMS data. The UV and IR data indicated that compound **2** was of the same type as compound **1**. The one-dimensional (1D) NMR data of compound **2** (Table 1) exhibited several signals (*δ*_H_ 5.13, 2.47, 2.22, and 1.61; *δ*_C_ 135.9, 124.8, 43.8, 23.1, and 16.0) that did not appear in compound 1. The COSY correlations from H-14 (*δ*_H_ 5.13) to H_2_-16 (*δ*_H_ 2.47) as well as HMBC correlations of H_3_-19 (*δ*_H_ 1.61)/C-12 and C-14 and H_3_-18 (*δ*_H_ 2.09)/C-16 implied that the additional linear fragment was present at C-12 (Figure 2). The other ^1^H and ^13^C NMR data for compound **2** were similar to those of compound **1**. The overall structure was completed by the other COSY and HMBC data. The ROESY correlations of H_2_-4/H-10 and H_2_-12/H-14 (Figure S13) suggested that the geometry of the double bonds was 3*Z* and 13*E*, respectively. Compound **2** was tentatively named sargassumin B.

### 2.3. Absolute Configuration Determination of Compounds

The absolute configuration at C-6 in compounds **1** and **2** was deduced by applying the octant rules. The circular dichroism (CD) data of compounds **1** and **2** exhibited negative cotton effects (CEs) of around 245 nm. These negative cotton effects could be explained by the carbonyl projection of the 6*R* model for the octant rule, and previous studies showed the same results (Figure 3B) [11]. Furthermore, the dimethyl carbinol group should be located in an equatorial position on the *δ*-lactone chair form due to 1,3-diaxial interactions. Therefore, compounds **1** and **2** were determined as 6*R*-(2-hydroxypropan-2-yl)-3*Z*-(4-oxopentylidene)tetrahydro-2H-pyran-2-one and 6*R*-(2-hydroxypropan-2-yl)-3*Z*-((13*E*)-4-methyl-8-oxonon-4-en-1-ylidene)tetrahydro-2H-pyran-2-one, respectively.

### 2.4. MAO Activities and Molecular Docking of the Isolated Compounds

Compound **1** was evaluated for its inhibitory effects against *h*MAO-A and *h*MAO-B enzymes. Compound **1** showed weak activity (42.18 ± 2.68%) against *h*MAO-A at 200 μM but did not show any activity against *h*MAO-B. The IC_50_ value of the positive control (moclobemide) was 89.72 ± 2.56 µM (7.32 ± 5.08% at 4 µM, 22.96 ± 3.40% at 20 µM, and 54.00 ± 1.57% at 100 µM). IC_50_ values of moclobemide varied according to the experiments used in the previous papers [12,13]. Although compound **2** was not tested due to insufficient quantity, compound **2** was inferred to be likely active as well, considering the structural similarity of these compounds.

**Figure 4:**
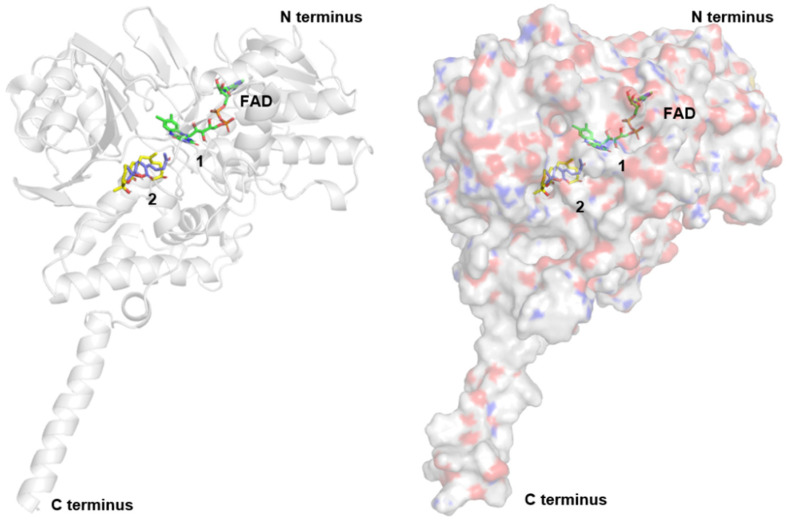
Binding model of compounds **1** and **2** in human monoamine oxidases A crystal structure (Protein Data Bank ID: 2Z5X).

**Figure 5:**
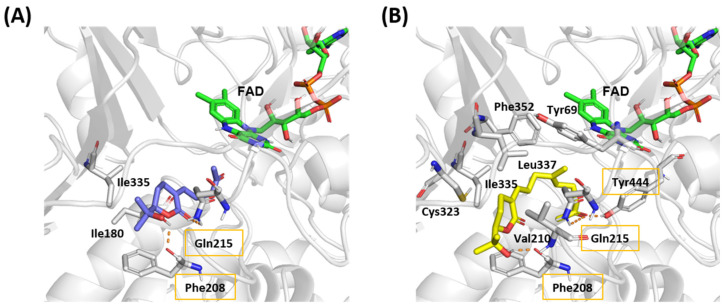
Stereoview of molecular docking poses of compounds **1** (**A**) and **2** (**B**). *h*MAO-A is shown as a white cartoon and carbon atoms of interacting residues are shown as white sticks. The carbon atoms in docking conformations of two compounds in *h*MAO-A are shown in blue and yellow sticks, respectively. Carbon atoms of flavin adenine dinucleotide (FAD) are shown as green sticks. FAD was considered as part of a protein during molecular docking. Hydrogen bond interactions are shown as orange dotted lines, and residues that participate in them are labeled with orange boxes. Residues that participate in hydrophobic interactions are labeled without boxes.

The molecular dockings of compounds **1** and **2** into *h*MAO-A were performed to indirectly check the feasibility of our inference (Figure 4 and Figure 5). The binding affinity scores of compounds **1** and **2** were −8.48 and −9.72 kcal/mol, respectively. The docking results suggested that these compounds formed hydrogen-bonding interactions with the oxygen backbone in Phe208 and nitrogen of the side-chain in Gln215. Furthermore, compound **1** participated in hydrophobic interactions with Ile180 and Ile335 that were already observed between *h*MAO-A and harmine in the crystal structure [14]. In particular, Ile335 has been known to play a role in the selectivity of *h*MAO-A [15]. To compare the binding ability of compound **1** to *h*MAO-A and *h*MAO-B, the molecular docking of compound **1** to *h*MAO-B was also performed. As seen in Figure S16, Tyr326* in *h*MAO-B (corresponding to Ile335 in *h*MAO-A) may prevent the binding of compound **1** and hinder the key hydrogen bond interaction between the backbone oxygen of Ile199* (corresponding to Phe208 in *h*MAO-A) and compound **1** as observed between Phe208 and compound **1** in *h*MAO-A.

Compound **2** also showed interaction with Phe208, Gln215, and Ile335. These three common interactions in both compounds implied that compound **2** could also exhibit actual activity against *h*MAO-A. Although compound **2** did not show interaction with Ile180, it showed interactions with Tyr69, Val210, Cys323, Leu337, and Phe352, suggesting that these additional interactions might be the reason for the higher affinity score of compound **2** compared to compound **1**. Furthermore, an additional hydrogen bond was observed with the side chain of Tyr444 that was not seen in the binding model of compound **1** or harmine bound crystal structure. Tyr444 has been known to play a role in the catalytic activity of *h*MAO-A [16]. Therefore, it is worth conducting a biological evaluation of compound **2**.

## 3. Discussion

In general, algae have been used in nutritional supplements for minerals, vitamins, proteins, and amino acids. Recently, there has been a growing interest in algae due to their unique composition. Several reports have suggested that algae are rich sources of various types of secondary metabolites [2]. The harsh environment in which algae live generally promotes the formation of various oxidizing agents and secondary metabolites including halogenated compounds, alcohols, aldehydes, sterols, and terpenoids [17]. These molecules are responsible for specific biological activities, such as antibacterial, antifungal, and growth-enhancing effects, that are effective in improving the survivability of algae [18].

In particular, brown algae possess fucoxanthin pigments and several tannins that give them characteristic greenish–brown colors [19]. Furthermore, these algae contain many biologically active compounds consisting of phenolic compounds, polysaccharides, and sterols [2]. Polysaccharides such as alginate and fucoidans have been reported to possess anticancer, antiviral, anti-inflammatory, and antiproliferative properties [18]. Sterols such as fucosterol have anticancer, cholesterol-reducing, and antidiabetic properties [20]. So far, more than 1140 compounds have been reported in brown algae [21]. Furthermore, according to the chemical database such as Reaxys^®^, approximately 500 different molecules have been reported in the brown algae *Sargassum* sp. Although a considerable number of molecules have been reported in these algae, only a few compounds including sterols, terpenoids, and furan derivatives, have been reported in *S. macrocarpum*. These characteristics suggest the need for in-depth research on algae.

The most well-known terpenoids in *Sargassum* sp. are sargachromenols. These compounds are categorized as meroterpenoids that possess a polyprenyl chain attached to quinone or aromatic rings. A considerable number of these compounds have chromene groups, which could exhibit cytotoxicity and antioxidant activity [10]. Previous studies have reported three meroterpenoids, sargaquinoic acid, sargahydroquinoic acid, and tuberatolide B, from *S. macrocarpum*, which showed neuroprotection, attenuation of inflammation, and the suppression of cancer progression, respectively [22,23,24]. These compounds also have quinone or aromatic rings attached to a polyprenyl chain. In the present study, we obtained two new terpenoids, sargassumins A and B (**1** and **2**), from *S. macrocarpum*. These compounds also have prenyl chains but consist of a monocyclic lactone group, unlike the above meroterpenoids. Terpenoids with lactone moieties have been shown to possess a broad spectrum of bioactivities [25]. Hence, further research to discover uses for these types of terpenoids is worth conducting.

Human monoamine oxidases A and B (*h*MAO-A and *h*MAO-B) are enzymes that catalyze the oxidative deamination of monoamine neurotransmitters and dietary amines. Recently, the inhibition of these enzymes is one of the effective strategies to discover molecules for the treatment of psychiatric or neurological diseases. So far, several molecules (isocarboxazid, phenelzine, selegiline, and tranylcypromine) have been clinically approved worldwide [7]. Among them, the selective inhibition of *h*MAO-A is an effective approach for the treatment of psychiatric disorders, while selective *h*MAO-B inhibitors are those used for treating Parkinson’s disease [8]. In particular, selective *h*MAO-A inhibitors such as the irreversible inhibitor clorgyline and the reversible inhibitor moclobemide are used in the treatment of depression and anxiety [8]. Even though considerable research effort has been expended, it is still worthwhile to discover molecules that potently inhibit these enzymes, given the beneficial role of these inhibitors.

Although there have been no reports that *Sargassum* sp.-derived extracts or compounds have MAO activities, a considerable number of reports have suggested that algae-derived products have the potential to possess these activities [9,26]. In this study, the 70% ethanol extract of *S. macrocarpum* inhibited both *h*MAO-A and *h*MAO-B. We used the Sepbox system for the bioassay-guided isolation due to its efficiency in enabling the rapid identification of biologically active compounds. Thus, we found that compound **1** has mild *h*MAO-A inhibition. Furthermore, the binding of compound **1** to the *h*MAO-A enzyme was deduced by molecular docking using a crystal structure. In the case of compound **2**, we could not evaluate the inhibitory effect against *h*MAO-A due to insufficient quantity. However, the binding affinities of compound **2** through several interactions implied that this compound could also have inhibitory activity. Although the predicted results of molecular docking do not perfectly match the actual results, three common interactions for both compounds **1** and **2** with *h*MAO-A amino acids suggest that further research to obtain a greater quantity of compound **2** is needed. Further research to find the active compound against *h*MAO-B is also needed.

## 4. Materials and Methods

### 4.1. Plant Material and Extraction

The leafy thalli of *Sargassum macrocarpum* C. Agardh (SMC) were collected by hand at the subtidal zone (0–2 m) on the southwestern shore of Jeju Island, Korea, in October 2018 and authenticated by Prof. H. R. Kim (Pukyong National University, Busan, Korea). A voucher specimen was deposited in the laboratory of Prof. J. S. Choi (Pukyong National University, Busan, Korea). The fresh *S. macrocarpum* was immediately frozen and kept at −25 °C. The sample was lyophilized (dry weight, 57 g) and extracted two times by 70% ethanol in water (5 L) using reflux at 60 °C for 2 h, then evaporated in vacuo to obtain the ethanol (EtOH) extract (25 g).

### 4.2. General Experimental Procedures

Optical rotations were measured on a Jasco P-1020 polarimeter using a 1 cm cell. ECD spectra were recorded using an Applied Photophysics Chirascan plus a circular dichroism detector. IR spectra were obtained on a Thermo Nicolet iS10 spectrometer. The NMR spectra were obtained on a 600 MHz NMR Bruker system at the NCIRF (National Center for Interuniversity Research Facilities at Seoul National University). The automated HPLC/SPE/HPLC coupling separation was performed on a Sepiatec Sepbox 2D-250 equipped with UV and ELSD detectors. HPLC-MS data were obtained from an Agilent 1200 system connected with 6120 quadrupole MSD equipped with a Phenomenex Luna C_18_ column (150 × 4.6 mm, 5 μm). Preparative (prep) HPLC was performed by a Gilson 321 HPLC with a UV/VIS-151 detector (210 nm) and a Shodex-RI-101 refractive index detector.

### 4.3. Sepbox Application for Subfraction of S. macrocarpum

The EtOH extract was partitioned using methylene chloride (CH_2_Cl_2_) and 10% ethanol in water. The CH_2_Cl_2_ layer (18.9 g) was subjected to a silica gel column eluted with a CH_2_Cl_2_–MeOH mixture solvent (50:1) as a mobile phase to produce 9 subfractions (SMC1–SMC9). Four (SMC3 to SMC6) fractions were combined in equal weights by 375 mg to make a total of 1.5 g of Sepbox sample. The combined sample was absorbed onto C_4_ reverse-phase (RP) resin (4.5 g) followed by subjection to the Sepbox system using the separation methods shown in Table S2. In total, 12 solid-phase extraction traps (SMC3456-1 to SMC3456-12) were collected through the C_4_ RP column with methanol and water mobile phase. Each hydrophilic trap (SMC3456-1 to SMC3456-4) was subjected to the second C_18_ Aqueous RP column (250 × 10 mm, 10 μm) with a flow rate of 3 mL/min. Five traps (SMC3456-5 to SMC3456-10) were subjected to the C_18_ RP column (250 × 10 mm, 10 μm) with a flow rate of 3 mL/min. Hydrophobic traps (SMC3456-11 and SMC3456-12) were subjected to the C_8_ RP column (250 × 10 mm, 10 μm) with a flow rate of 3 mL/min. After the second HPLC separation, samples were collected into the 48-well plate. All the 288 wells were combined into a total of 55 vials under the guidance of the UV and ELSD chromatograms.

### 4.4. Purification of Compounds ***1*** and ***2***

SMC3456-4-2 was purified by prep-HPLC (acetonitrile–H_2_O with 0.02% trifluoroacetic acid, 2:3 to 59:41 in 54 min, 3 mL/min) with a Luna C_18_ column (250 × 10.0 mm, 10 μm) to obtain compound **1** (1.7 mg, *t*_R_ 10 min). SMC3456-9-3 was purified by prep-HPLC (acetonitrile–H_2_O, 3:1, 2.5 mL/min) with YMC-Pack ODS-A column (250 × 10.0 mm, 5 μm) to obtain compound **2** (0.8 mg, *t*_R_ 12.0 min) with an RI detector.

#### 4.4.1. Sargassumin A (1)

Colorless oil; [*α*]^20^_D_ −129.7 (*c* 0.1, MeOH); UV (MeOH) *λ*_max_ (log *ε*) 225 (4.60) nm; CD (*c* 2.1 × 10^−3^ M, acetonitrile) (Δ*ε*) 246 (−0.06), 285 (−0.08) nm; IR (*ν*_max_) 3422, 2921, 2856, 2850, 1695, 1463, 1380, 1133 cm^−1^; HRESIMS m/z 241.1429 [M + H]^+^ (calculated for C_13_H_21_O_4_, *m*/*z* 241.1440); ^1^H and ^13^C NMR data in Table 1.

#### 4.4.2. Sargassumin B (2)

Colorless oil; [*α*]^20^_D_ +113.9 (*c* 0.1, MeOH); UV (MeOH) *λ*_max_ (log *ε*) 218 (4.75) nm; CD (*c* 3.2 × 10^−3^ M, ethanol) (Δ*ε*) 245 (−0.07), 267 (−0.06) nm; IR (*ν*_max_) 3437, 2891, 2848, 1697, 1443, 1389, 1140 cm^−1^; HRESIMS *m*/*z* 309.2055 [M + H]^+^ (calculated for C_18_H_29_O_4_, *m*/*z* 309.2066); ^1^H and ^13^C NMR data in Table 1.

### 4.5. hMAO Assay of Compounds ***1*** and ***2***

The *h*MAO bioassay was carried out based on a previous report using the MAO-Glo™ assay kit (Promega, Madison, WI, USA). The readymade buffer solution was used as diluting solvent for enzyme preparation and sample preparation. The luciferin detection agent comes with the solution itself, so there is no need to prepare a separate solution.

Briefly, 12.5 μL of test compounds or a positive compound (l-deprenyl) were added to 12.5 μL of luciferin derivative substrate (160 and 16 μM of *h*MAO-A and *h*MAO-B, respectively) in each well of the plate. The 25 μL of enzyme solution was added to the compounds to initiate the reaction and the plate was incubated at 25 °C for 1 h. Then, 50 µL of reconstituted luciferin detection reagent was added to stop the *h*MAO reaction and the plate was incubated at 25 °C for 5 min. Luminescence was measured on a FilterMax F5 Multi-Mode microplate reader (Molecular Devices, San Jose, CA, USA). The percentage of inhibition of the test compounds has been calculated by the formula: (luminescence of blank-luminescence of sample)/luminescence of blank × 100.

### 4.6. Structural Preparation and Molecular Docking

The X-ray crystal structures of *h*MAO-A bound to harmine and *h*MAO-B bound to safinamide were obtained from the Protein Data Bank (accession code: 2Z5X) [14]. The ligand, co-crystallized water molecules, organic solvents, and ions were removed except for the cofactor flavin adenine dinucleotide. The resulting protein structure was prepared in Discovery Studio client (DSC) v19.1 by inserting missing loops using a modeler, energy minimization, and protonation.

The molecular docking was performed in AutoDock 4.2, and the ligand and protein structures for docking were prepared using AutoDockTools 1.5.6. The grid center was set using harmine or safinamide, and 1000 runs with the Lamarckian genetic algorithm were given for the ligand conformational search. The grid and docking parameter files were prepared using Python and C Shell scripts provided in the AutoDock tutorial. The best pose from the docking clusters was chosen and interactions were analyzed in DSC v19.1. Figures were generated in PyMol. The molecular docking procedure was validated by re-docking harmine in the crystal structure (Figure S15). Figures were generated in PyMol.

## 5. Conclusions

A chemical investigation of brown algae *S. macrocarpum* resulted in the identification of two new terpenoid lactones (**1** and **2**). Furthermore, the biological investigation demonstrated that compound **1** had mild inhibitory activity against the *h*MAO-A enzyme. In addition, compound **1** docked computationally into the active site of *h*MAO-A. Although compound **2** could not be tested due to insufficient quantity, compound **2** docked better into *h*MAO-A. This implies that it is likely that there are other potent molecules into *S. macrocarpum* and therefore further detailed chemical investigations are needed. Consequently, it is envisaged that this study will contribute to a better understanding of the phytochemistry of the brown algae *S. macrocarpum* and will contribute to the discovery of the potential lead compounds for the treatment of several psychiatric or neurological diseases.

## Figures and Tables

**Table 1:** ^1^H (600 MHz) and ^13^C (150 MHz) nuclear magnetic resonance data of compounds **1** and **2**.

Position	1 (Chloroform-*d*)		2 (Acetone-*d*_6_)	
	*δ*_C_	*δ*_H_	*δ*_C_	*δ*_H_
1				
2	165.2		165.7	
3	125.3		126.8	
4	28.6	2.58, m	29.4	2.56, m
		2.53, m		
5	23.4	1.99, ddt (13.5, 5.5, 2.5)	24.2	2.08, m
		1.71, dtd (13.5, 12.0, 6.0)		1.68, dtd (13.5, 11.5, 7.0)
6	85.8	4.04, dd (12.0, 3.0)	86.3	4.01, dd (12.0, 3.0)
7	71.4		71.2	
8	24.2	1.23, s	25.4	1.20
9	25.7	1.29, s	26.5	1.20
10	146.0	6.13, br tt (7.5, 2.0)	146.1	6.03, br tt (7.0, 2.0)
11	24.0	2.83, br dtt (7.0, 7.0, 1.0)	28.5	2.66, dtt (7.0, 7.0, 1.0)
12	42.8	2.63, br t (7.0)	39.6	2.08, m
13	208.1		135.9	
14	29.7	2.16, s	124.8	5.13, br tq (7.0, 1.0)
15			23.1	2.22, dt (7.0, 7.0)
16			43.8	2.47, t (7.0)
17			207.8	
18			30.7	2.09, s
19			16.0	1.61, s

## Data Availability

Not applicable.

## Supplementary Materials

The following are available online at https://www.mdpi.com/article/10.3390/plants11151998/s1. Figures S1–S16: The ^1^H, ^13^C, COSY, HSQC, HMBC, ROESY NMR, and HRMS spectra of compounds **1** and **2**, docking of harmine in *h*MOA-A, and Stereoview of specific molecular docking poses of compounds **1** into *h*MOA-A and *h*MOA-B; Tables S1 and S2: *h*MAO inhibitory activity of ethanol extract and fractions from *S. macrocarpum* and the fractionation conditions of the methylene chloride subfraction of *S. macrocarpum* using the Sepbox system.
