# Supplementary material for: New Monocyclic Terpenoid Lactones from a Brown Algae Sargassum macrocarpum as Monoamine Oxidase Inhibitors
Source: Plants (Basel). 2022 Jul 31;11(15):1998. doi: 10.3390/plants11151998 (PMC9370394; doi:10.3390/plants11151998)
Supplement: Supplementary file 1 [file plants-11-01998-s001.zip › plants-1786834-supplementary.pdf]

plants

MDPI

Article

New Monocyclic Terpenoid Lactones from a Brown Algae *Sargassum macrocarpum* as Monoamine Oxidase Inhibitors

Jaeyoung Kwon [1,2,†], Kyerim Lee [1,3,†], Hoseong Hwang [1,4,†], Seong-Hwan Kim [1], Se Eun Park [5], Prasannavenkatesh Durai [1], Keunwan Park [1], Hyung Seop Kim [4], Dae Sik Jang [3,6], Jae Sue Choi [7,*], Hak Cheol Kwon [1,3,*]

List of supporting information

Figure S1. ^{1}H NMR spectrum of compound **1** in chloroform-*d*.
Figure S2. ^{13}C NMR spectrum of compound **1** in chloroform-*d*.
Figure S3. COSY NMR spectrum of compound **1** in chloroform-*d*.
Figure S4. HSQC NMR spectrum of compound **1** in chloroform-*d*.
Figure S5. HMBC NMR spectrum of compound **1** in chloroform-*d*.
Figure S6. ROESY NMR spectrum of compound **1** in chloroform-*d*.
Figure S7. The HRESIMS spectrum of compound **1**.
Figure S8. ^{1}H NMR spectrum of compound **2** in acetone-d_6.
Figure S9. ^{13}C NMR spectrum of compound **2** in acetone-d_6.
Figure S10. COSY NMR spectrum of compound **2** in acetone-d_6.
Figure S11. HSQC NMR spectrum of compound **2** in acetone-d_6.
Figure S12. HMBC NMR spectrum of compound **2** in acetone-d_6.
Figure S13. ROESY NMR spectrum of compound **2** in acetone-d_6.
Figure S14. The positive HRESIMS spectrum of compound **2**.
Figure S15. Docking of harmine in *h*MOA-A (PDB id: 2Z5X) to validate the molecular docking procedure. Carbon atoms in crystal bound harmine and docked harmine are shown as pale yellow and light magenta sticks, respectively (RMSD 0.61 Å).
Figure S16. Stereoview of molecular docking poses of compounds **1**. Human monoamine oxidases A and B (*h*MAO-A and *h*MAO-B) are shown as white cartoons and carbon atoms of interacting residues are shown as white sticks and orange sticks, respectively. Asterisks represent *h*MAO-B residues. The carbon atoms in docking conformations of compound **1** in *h*MAO-A and hMAO-B are shown in blue and cyan sticks, respectively. FAD's carbon atoms are shown as green sticks. FAD was considered as a part of protein during molecular docking. Hydrogen bond interactions are shown as orange dotted lines.
Table S1. *h*MAO inhibitory activity of ethanol extract and fractions from *S. macrocarpum*
Table S2. The fractionation conditions of the methylene chloride subfraction of *S. macrocarpum* using the Sepbox system.

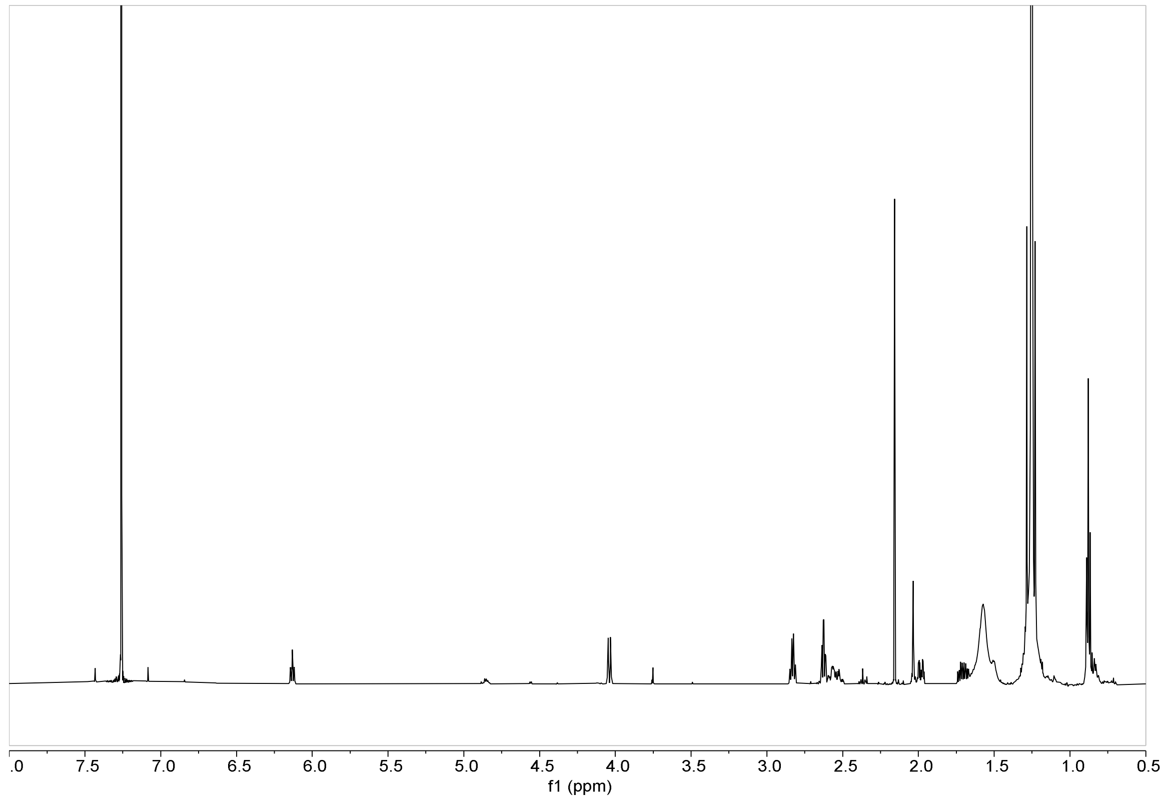

Figure S1. ^{1}H NMR spectrum of compound **1** in chloroform-*d*.

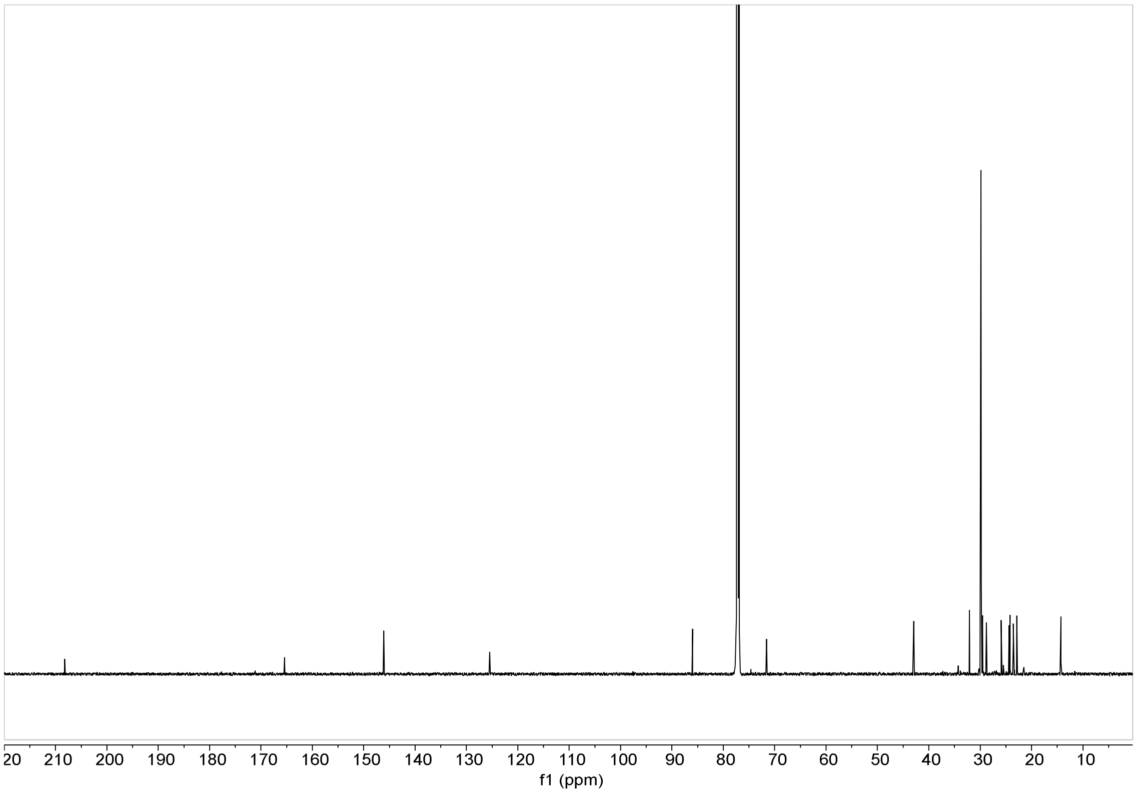

Figure S2. ^{13}C NMR spectrum of compound **1** in chloroform-*d*.

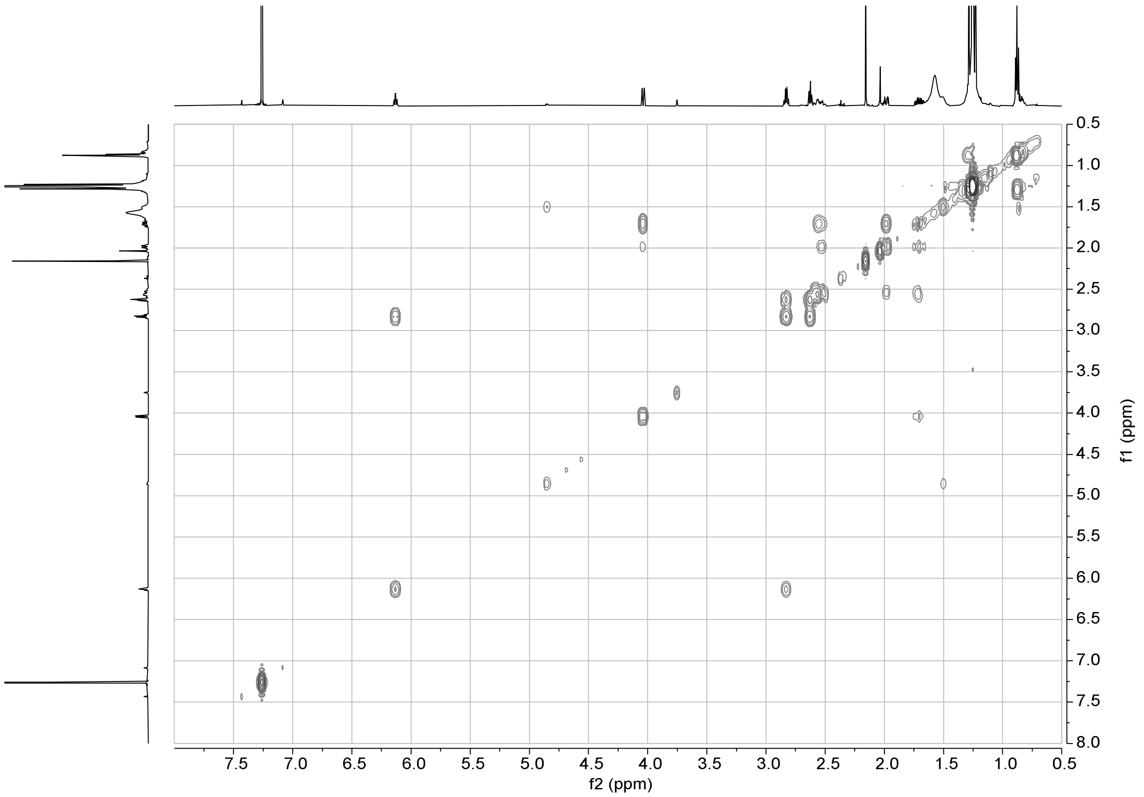

Figure S3. COSY NMR spectrum of compound **1** in chloroform-*d*.

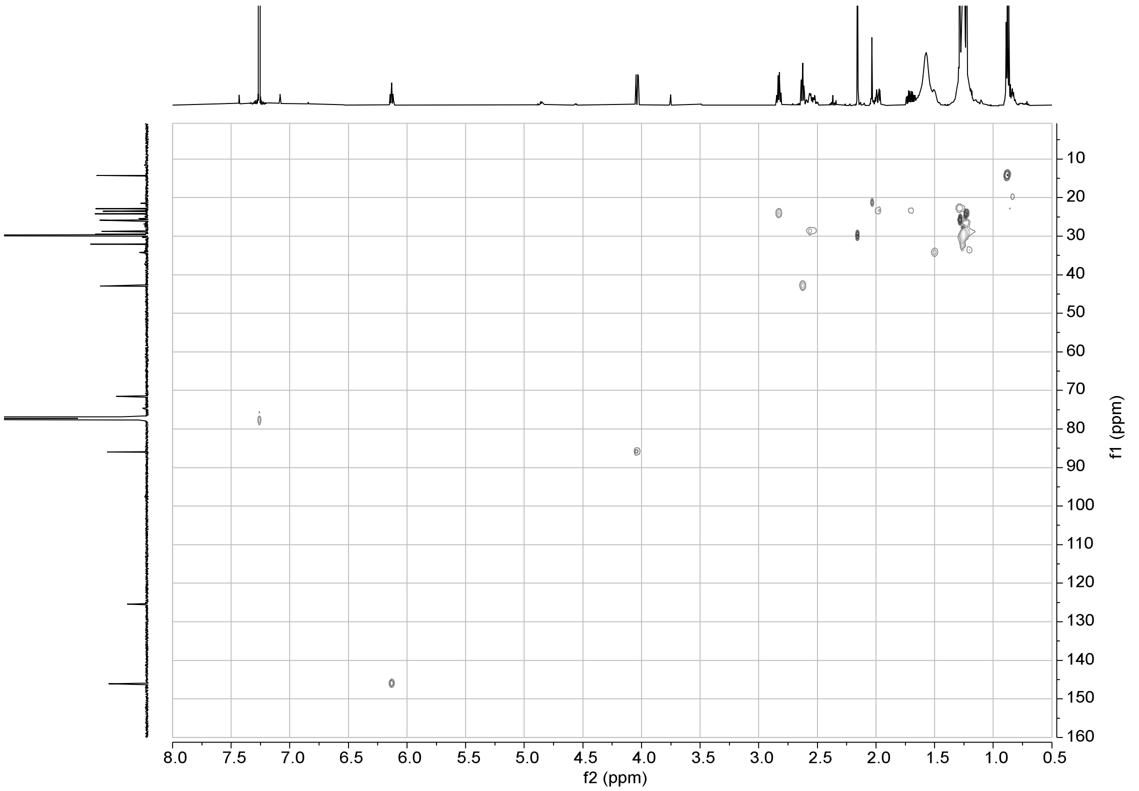

Figure S4. HSQC NMR spectrum of compound **1** in chloroform-*d*.

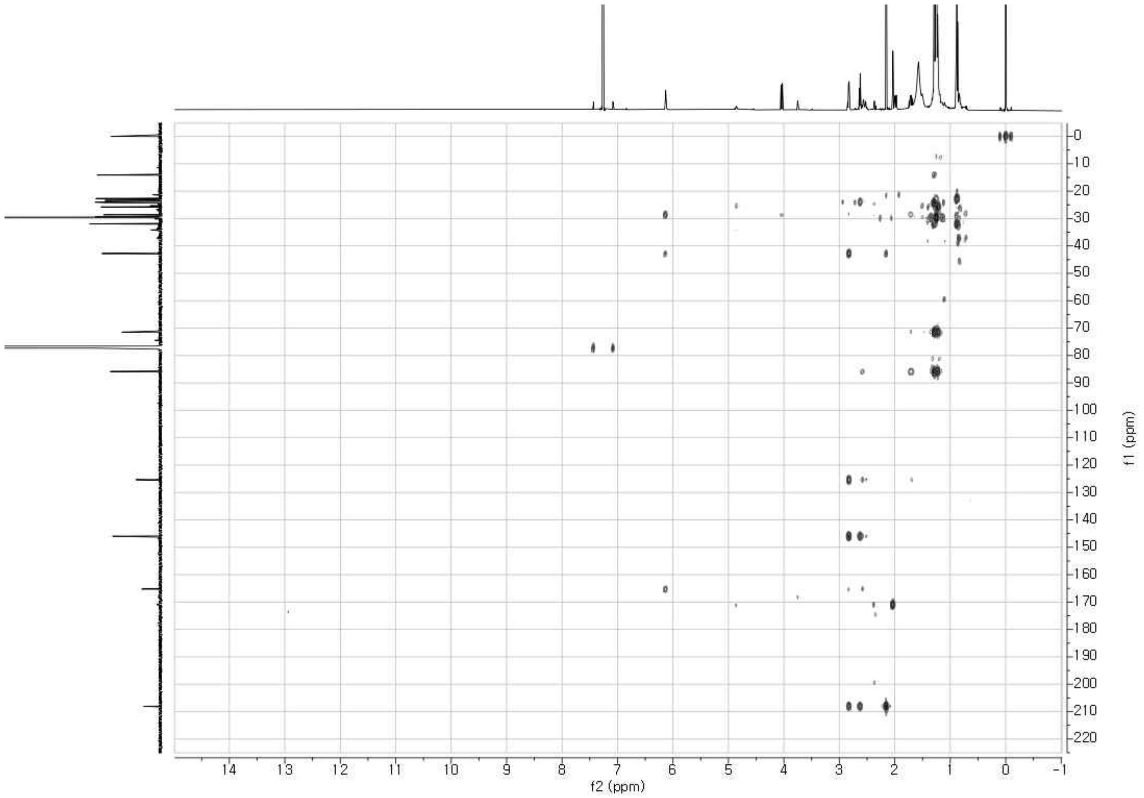

Figure S5. HMBC NMR spectrum of compound **1** in chloroform-*d*.

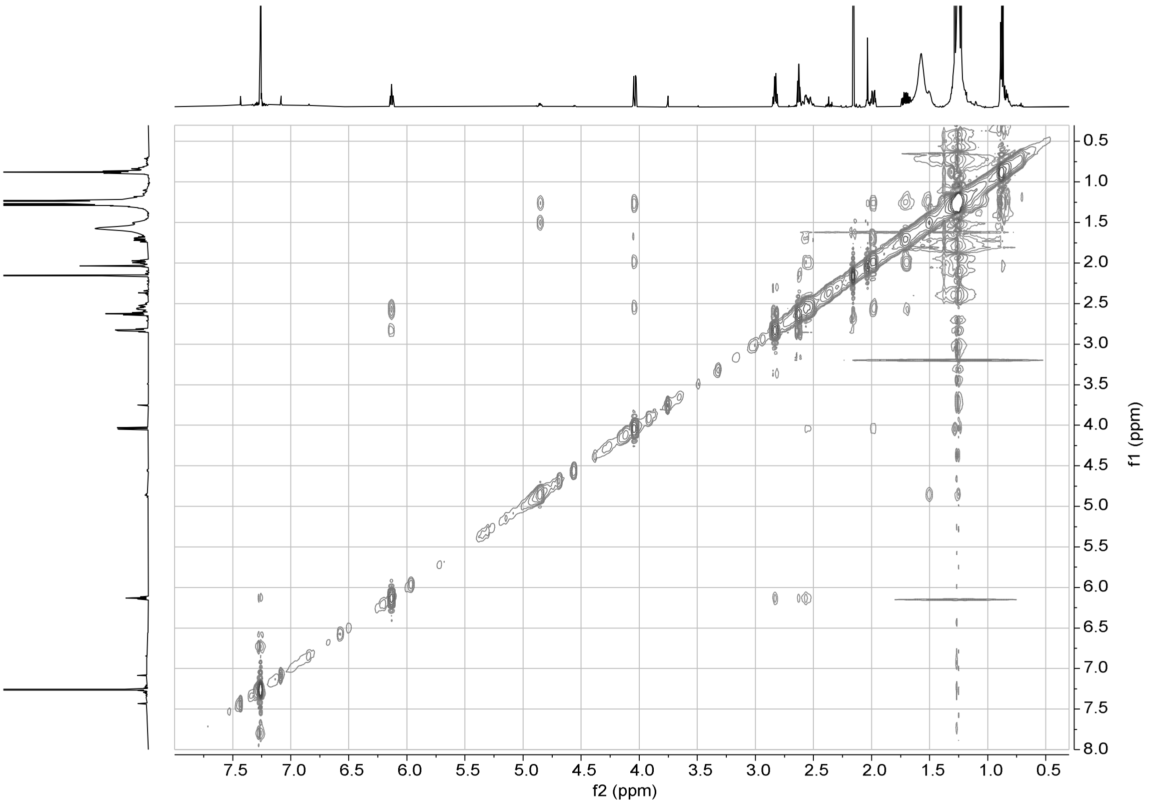

Figure S6. ROESY NMR spectrum of compound **1** in chloroform-*d*.

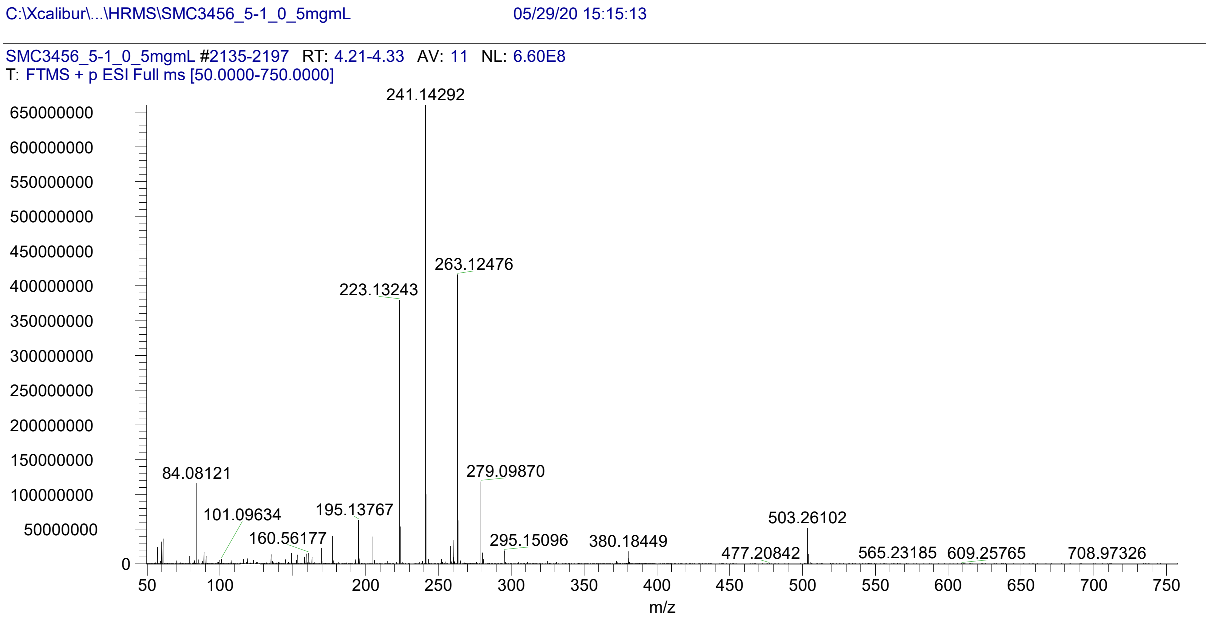

Figure S7. The HRESIMS spectrum of compound **1**.

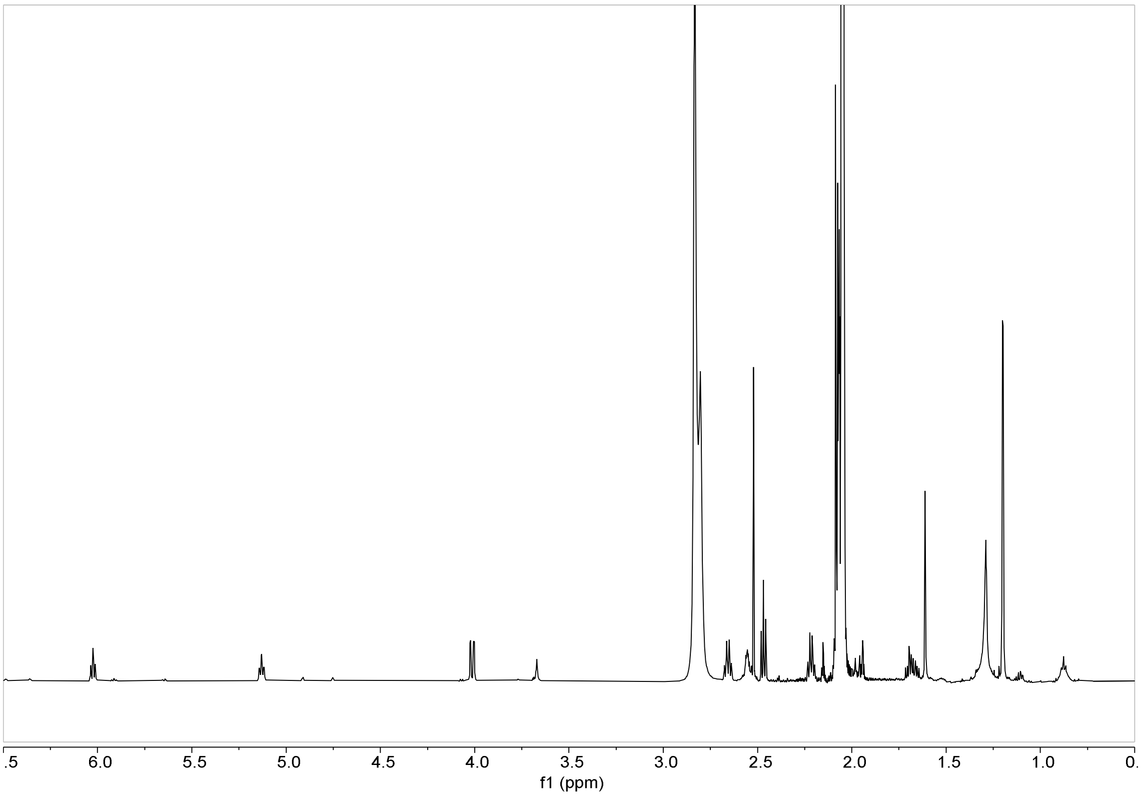

Figure S8. ^{1}H NMR spectrum of compound **2** in acetone-d_6.

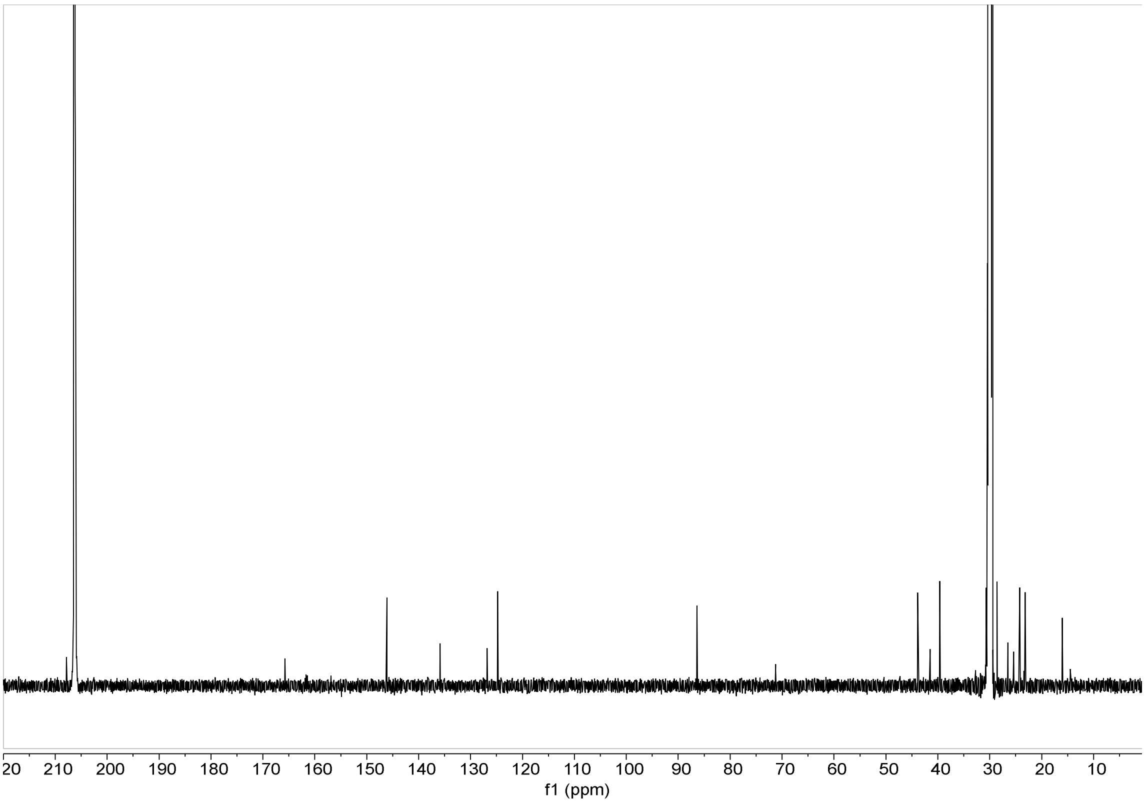

Figure S9. ^{13}C NMR spectrum of compound **2** in acetone-d_6.

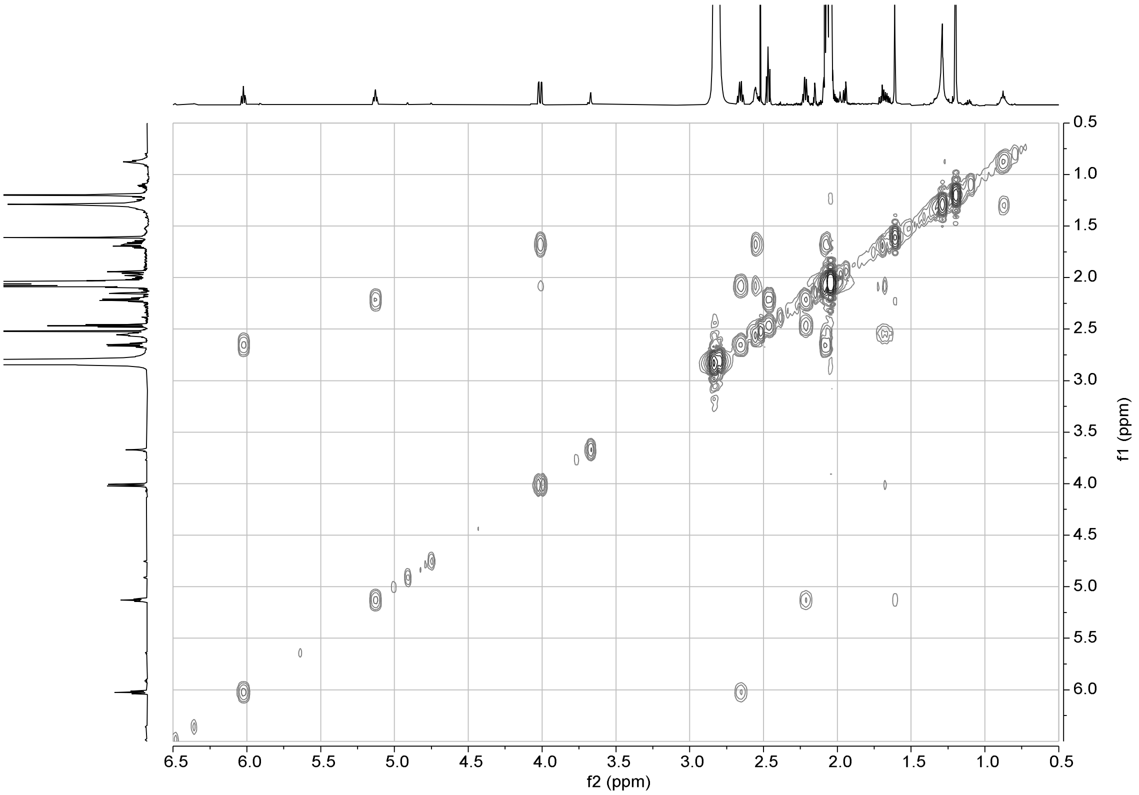

Figure S10. COSY NMR spectrum of compound **2** in acetone-*d*6.

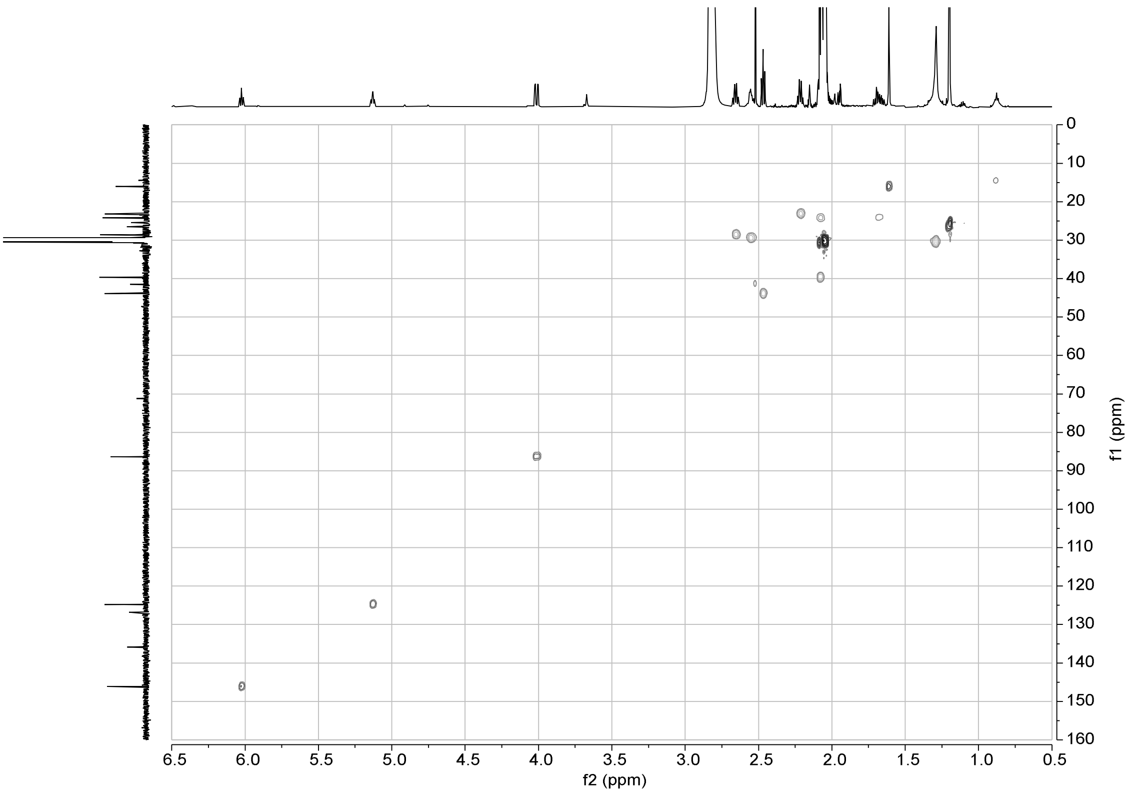

Figure S11. HSQC NMR spectrum of compound **2** in acetone-d_6.

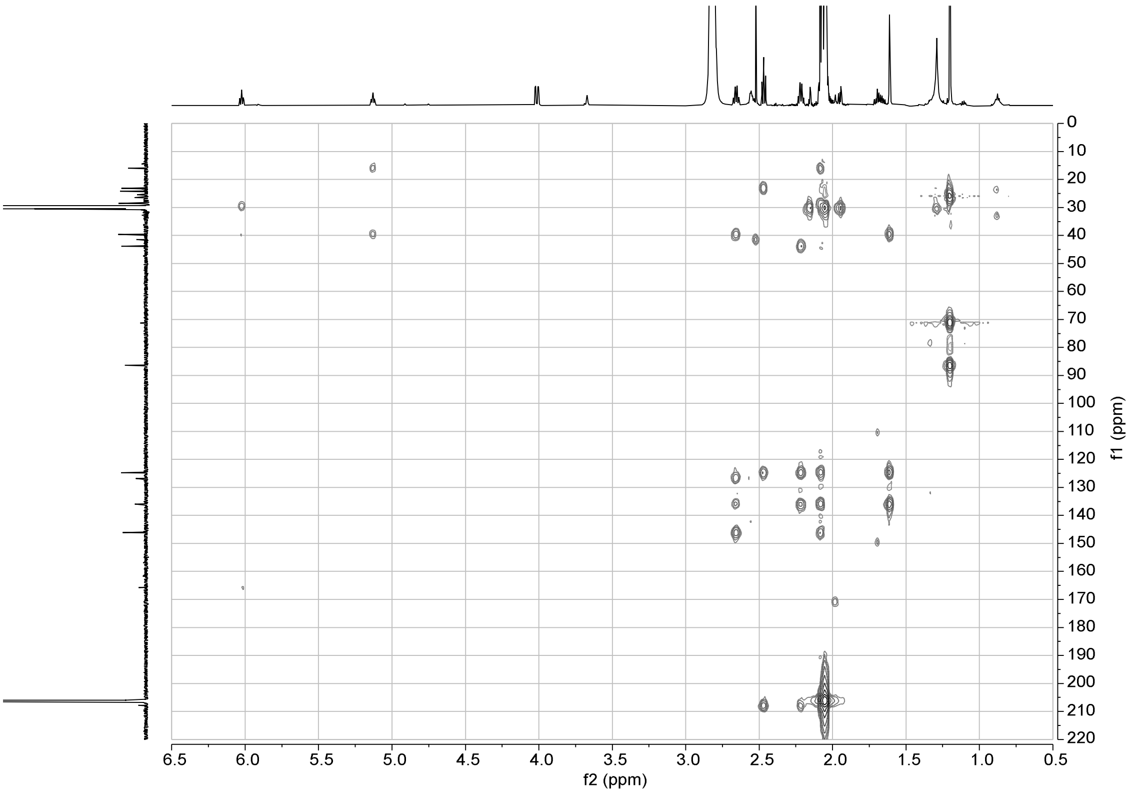

Figure S12. HMBC NMR spectrum of compound **2** in acetone-d_6.

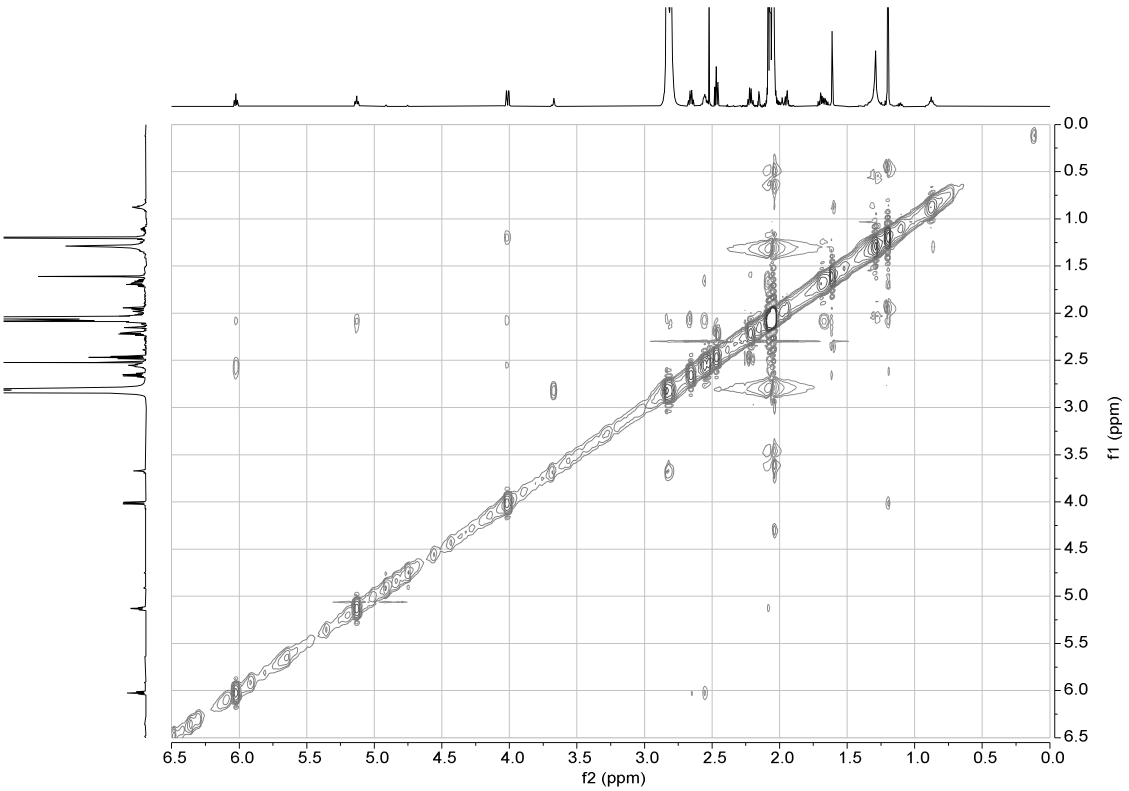

Figure S13. ROESY NMR spectrum of compound **2** in acetone-d_6.

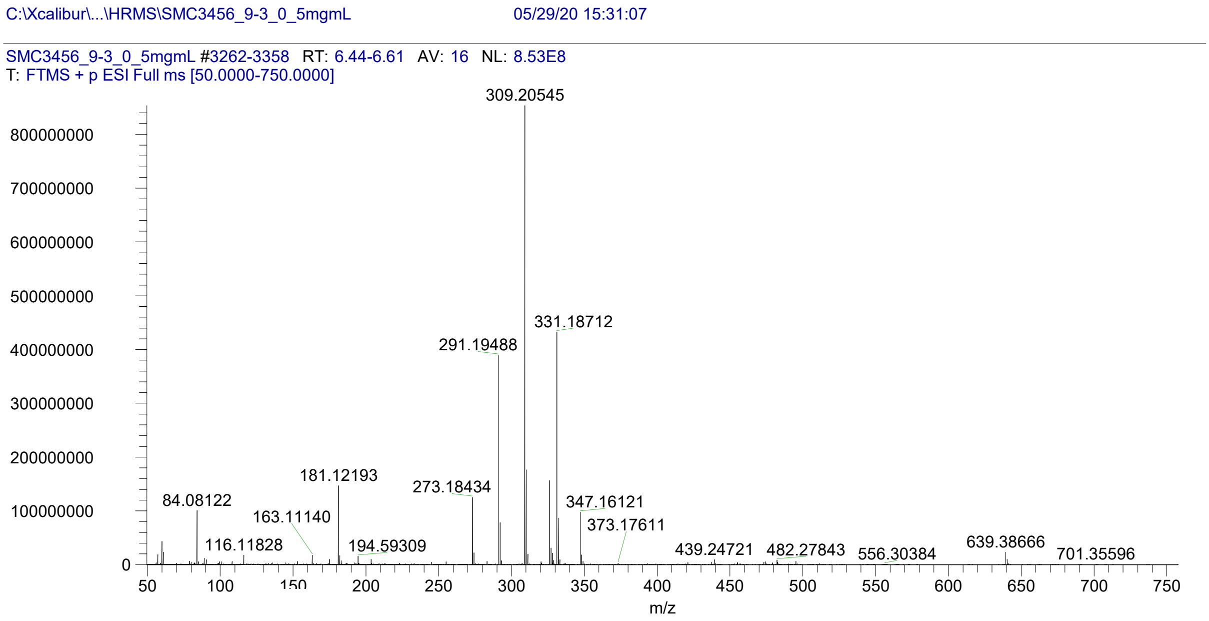

Figure S14. The HRESIMS spectrum of compound **2**.

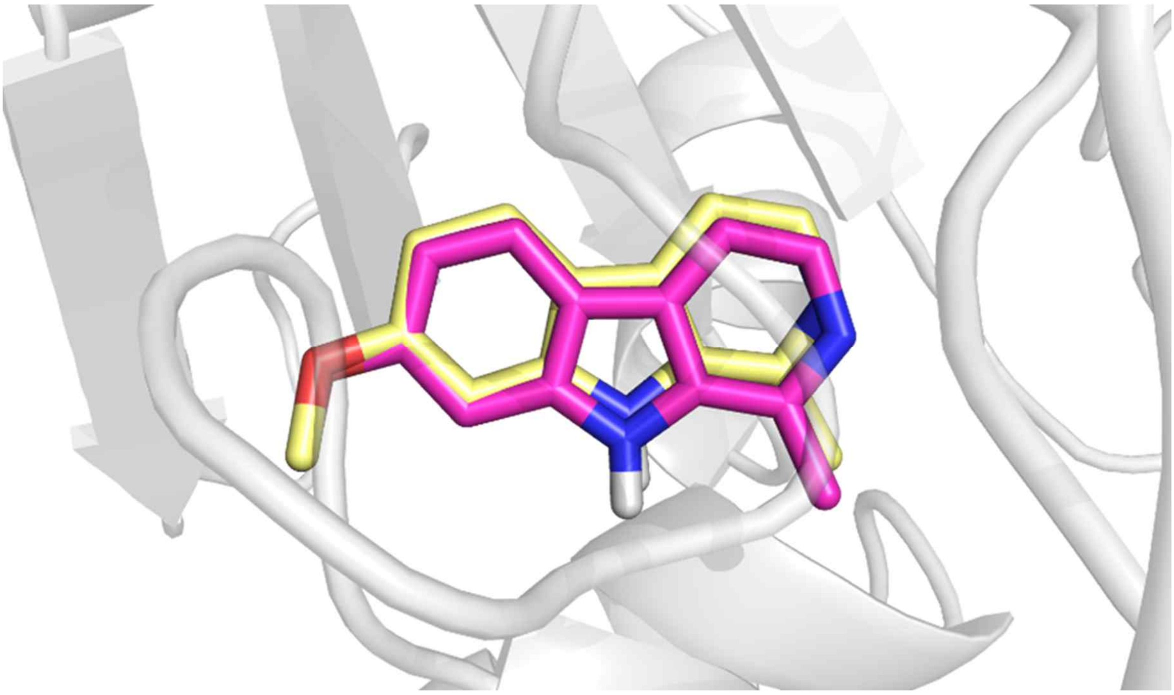

Figure S15. Docking of harmine in *h*MOA-A (PDB id: 2Z5X) to validate the molecular docking procedure. Carbon atoms in crystal bound harmine and docked harmine are shown as pale yellow and light magenta sticks, respectively (RMSD 0.61 Å).

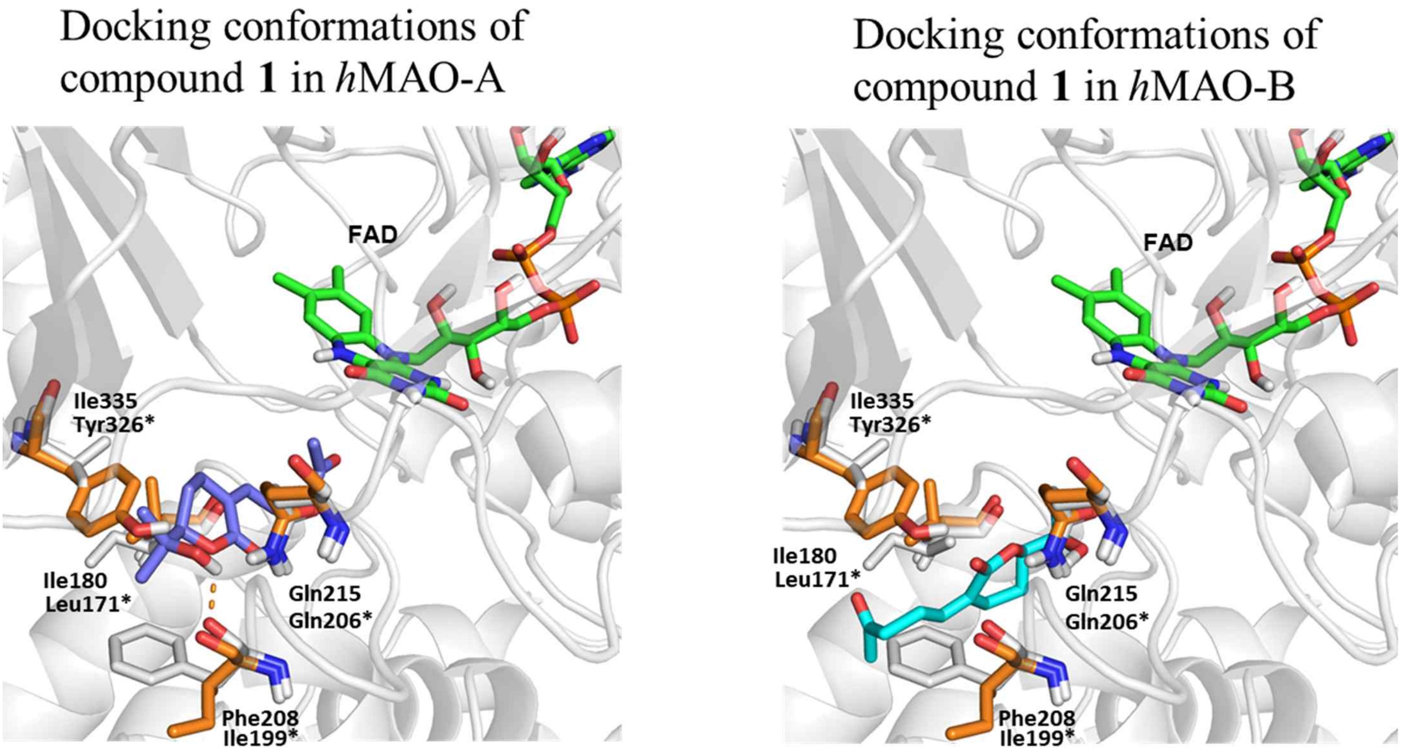

Figure S16. Stereoview of molecular docking poses of compounds **1**. Human monoamine oxidases A and B (*h*MAO-A and *h*MAO-B) are shown as white cartoons and carbon atoms of interacting residues are shown as white sticks and orange sticks, respectively. Asterisks represent *h*MAO-B residues. The carbon atoms in docking conformations of compound **1** in *h*MAO-A and hMAO-B are shown in blue and cyan sticks, respectively. FAD's carbon atoms are shown as green sticks. FAD was considered as a part of protein during molecular docking. Hydrogen bond interactions are shown as orange dotted lines.

Table S1. *h*MAO inhibitory activity of ethanol extract and fractions from *S. macrocarpum*

Sample	*h*MAO-A			*h*MAO-B		
	Conc. (μg/mL or μM)	Inhibition (%) (Mean ± SD)	IC_{50} value (μg/mL or μM ± SD) [b]	Conc. (μg/mL or μM)	Inhibition (%) (Mean ± SD)	IC_{50} value (μg/mL or μM ± SD) [b]
EtOH ext.	10	22.16 ± 5.61	103.15 ± 11.16	10	29.79 ± 2.69	80.41 ± 6.18
	40	31.84 ± 4.30		40	40.26 ± 3.44	
	200	78.13 ± 1.54		200	82.42 ± 2.34	
CH_2Cl_2 fr.	10	40.16 ± 1.27	21.37 ± 0.97	10	42.37 ± 0.67	17.03 ± 0.42
	40	66.07 ± 1.03		40	74.89 ± 0.92	
	200	94.48 ± 0.47		200	88.57 ± 0.81	
H_2O fr.	10	17.73 ± 2.65	>200	10	14.56 ± 4.22	>200
	40	15.58 ± 6.17		40	13.37 ± 4.72	
	200	18.22 ± 3.64		200	13.24 ± 1.91	
Moclobemide [a]	4	7.32 ± 5.08	89.72 ± 2.56	-	-	-
	20	22.96 ± 3.40		-	-	-
	100	54.00 ± 1.57		-	-	-
L-Deprenyl [a]	6.25	41.89 ± 2.40	8.42 ± 0.56	6.25	44.77 ± 3.27	0.12 ± 0.01
	12.5	65.20 ± 1.98		12.5	75.70 ± 2.10	
	25	78.75 ± 0.31		25	95.40 ± 1.30	

[a] Positive control was treated with the concentration value unit μM. [b] The 50% inhibitory concentration (IC_{50}) values were calculated from a dose-response curve and are expressed as the mean ± SD of triplicate experiments.

Table S2. The fractionation conditions of the methylene chloride subfraction of *S. macrocarpum* using the sepbox system.

1st separation		Elution time		Methanol/water	Results
	C_4 RP HPLC column (200 × 20 mm, 10 μm, 8 mL/min)	0-14	min	50/50	SMC3456-1 to 12
		14-44	min	80/20	
		44-65	min	100/0	
**2nd separation**		**Elution time**		**Acetonitrile/water**	**Results**
Step 1	SMC3456-1 (T 0-4 min)<br>C_{18} Aqueous RP HPLC column (250 × 10 mm, 10 μm)	0-4	min	5/95	No peak
		4-48	min	5/95-15/85	
		48-53	min	15/85-45/55	
		53-65	min	45/55	
Step 2	SMC3456-2 (T 4-8 min)<br>C_{18} Aqueous RP HPLC column (250 × 10 mm, 10 μm)	0-4	min	9/91	SMC3456-2-1 to 3<br>Vial 1-3
		4-48	min	9/91-24/76	
		48-53	min	24/76-48/52	
		53-65	min	48/52	
Step 3	SMC3456-3 (T 4-8 min)<br>C_{18} Aqueous RP HPLC column (250 × 10 mm, 10 μm)	0-48	min	13/87-33/67	SMC3456-3-1<br>Vial 4
		48-53	min	33/67-59/41	
		53-65	min	59/41	
Step 4	SMC3456-4 (T 4-8 min)<br>C_{18} Aqueous RP HPLC column (250 × 10 mm, 10 μm)	0-48	min	14/86-37/63	SMC3456-4-1 to 4<br>Vial 5-8
		48-53	min	37/63-63/37	
		53-65	min	63/37	
Step 5	SMC3456-5 (T 4-8 min)<br>C_{18} RP HPLC column (250 × 10 mm, 10 μm)	0-48	min	19/81-43/57	SMC3456-5-1<br>Vial 9
		48-53	min	43/57-67/33	
		53-65	min	67/33	
Step 6	SMC3456-6 (T 4-8 min)<br>C_{18} RP HPLC column (250 × 10 mm, 10 μm)	0-48	min	25/75-48/52	SMC3456-6-1 to 2<br>Vial 10-11
		48-53	min	48/52-71/29	
		53-65	min	71/29	
Step 7	SMC3456-7 (T 4-8 min)<br>C_{18} RP HPLC column (250 × 10 mm, 10 μm)	0-48	min	32/68-58/42	SMC3456-7-1 to 4<br>Vial 12-15
		48-53	min	58/42-78/22	
		53-65	min	78/22	
Step 8	SMC3456-8 (T 4-8 min)<br>C_{18} RP HPLC column (250 × 10 mm, 10 μm)	0-48	min	38/62-66/34	SMC3456-8-1 to 7<br>Vial 16-22
		48-53	min	66/34-85/15	
		53-65	min	85/15	
Step 9	SMC3456-9 (T 4-8 min)<br>C_{18} RP HPLC column (250 × 10 mm, 10 μm)	0-48	min	45/55-72/28	SMC3456-9-1 to 9<br>Vial 23-31
		48-53	min	72/28-92/8	
		53-65	min	92/8	
Step 10	SMC3456-10 (T 4-8 min)<br>C_{18} RP HPLC column (250 × 10 mm, 10 μm)	0-48	min	56/44-83/17	SMC3456-10-1 to 8<br>Vial 32-39
		48-53	min	83/17-100/0	
		53-65	min	100/0	
Step 11	SMC3456-11 (T 4-8 min)<br>C_8 RP HPLC column (250 × 10 mm, 10 μm)	0-48	min	60/40-87/13	SMC3456-11-1 to 11<br>Vial 40-50
		48-53	min	87/13-100/0	
		53-65	min	100/0	

Step 12	SMC3456-12 (T 4-8 min)<br>C_8 RP HPLC column (250 × 10 mm, 10 µm)	0-48	min	67/33-95/5	SMC3456-12-1 to 5<br>Vial 51-55
		48-53	min	95/5-100/0	
		53-65	min	100/0	
